# Hormones, receptors, and growth in hyperplastic enlarged lobular units: early potential precursors of breast cancer

**DOI:** 10.1186/bcr1367

**Published:** 2005-12-16

**Authors:** Sangjun Lee, Syed K Mohsin, Sufeng Mao, Susan G Hilsenbeck, Dan Medina, D Craig Allred

**Affiliations:** 1 Breast Center, Baylor College of Medicine, Houston, Texas, USA; 2 Department of Pathology, Baylor College of Medicine, Houston, Texas, USA; 3 Department of Molecular and Cellular Biology, Baylor College of Medicine, Houston, Texas, USA

## Abstract

**Introduction:**

The hyperplastic enlarged lobular unit (HELU) is a common alteration in adult female human breast and is the earliest histologically identifiable lesion with premalignant potential. Growth and differentiation in normal epithelium are regulated by estrogen and progesterone, whose effects are mediated through estrogen receptor (ER)-α and progesterone receptor (PR). We assessed correlations between growth (proliferation and apoptosis), endogenous hormone levels (using age as a surrogate for menopausal/estrogen status), and ER-α/PR expression in HELUs versus adjacent normal terminal duct lobular units (TDLUs) to gain insight into potentially premalignant hyperplasia.

**Methods:**

Proliferation (Ki67 antigen), ER-α, and PR were assessed by immunohistochemistry, apoptosis using the TUNEL (terminal transferase-mediated dUTP nick end-labeling) assay, and nuclear colocalization of ER-α and Ki67 by dual-labeled immunofluorescence in HELUs and adjacent TDLUs (*n *= 100–584, depending on the factor) from 324 breasts. All factors were quantified under direct microscopic visualization. ER-α/PR expression was semiquantified by estimating the proportion of positive cells (0 = none, 1 = <1/100, 2 = 1/100 to 1/10, 3 = 1/10 to 1/3, 4 = 1/3 to 2/3, and 5 = >2/3). Ki67, TUNEL, and colocalization of ER-α and Ki67 were scored by absolute counting (%positive).

**Results:**

ER-α and PR expression were significantly elevated in HELUs versus adjacent TLDUs (average score: 4.5 versus 3.1 and 3.5 versus 2.1; *P *< 0.0001). Proliferation was also significantly higher in HELUs versus TDLUs (average 6.3% versus 2.0%; *P *< 0.0001). In contrast, apoptosis was significantly lower in HELUs versus TDLUs (average 0.61% versus 0.22%; *P *< 0.0001). Changes in proliferation and receptor expression were similar between premenopausal and postmenopausal TDLUs and HELUs, suggesting that hyperplastic cells remain responsive to regulation by estrogen. The proportion of ER-positive/proliferating cells was much higher in HELUs than TDLUs (27.6% vs. 4.9%; *P* < .0001).

**Conclusion:**

Development of HELUs is associated with increased proliferation and decreased cell death relative to normal cells. ER-α and PR are highly elevated in HELUs, which may contribute to the hyperplasia because they mediate hormonal regulation of growth. An understanding of the fundamental causes of increased levels of receptors and growth may lead to new strategies to prevent breast cancer.

## Introduction

**Figure 1:**
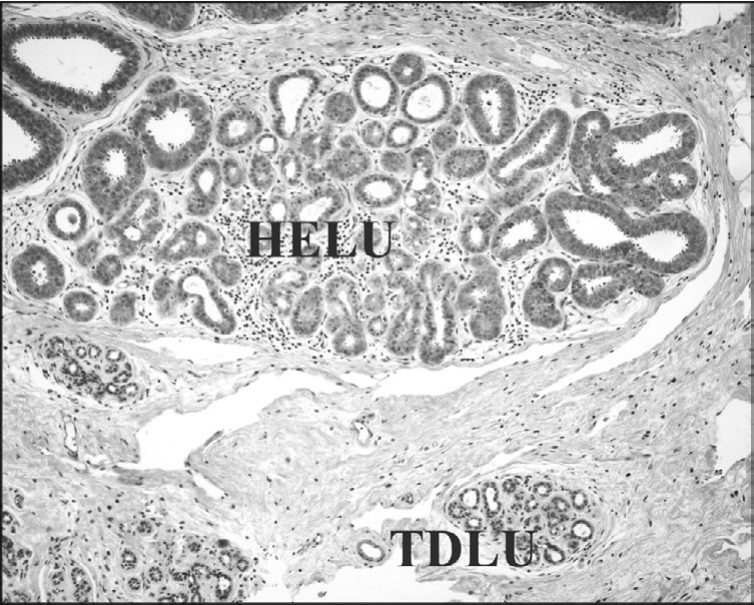
HELUs are much larger than normal TDLUs. This photomicrograph shows a normal type 1 TDLU adjacent to a typical HELU. In this example, the TDLU measures about 0.4 mm in diameter, contains about 150 cells within this area, and approximately 4000 total cells. The HELU measures about 2.0 mm in diameter, contains about 1500 cells within this area, and approximately 200,000 total cells (i.e. a 50-fold increase relative to the TDLU). Estimates of cell counts were based on calculations assuming all cells measure 10 μm in thickness and are evenly distributed within a sphere the diameter of the TDLU or HELU. Typical HELUs measure between 1 and 4 mm in diameter, and so the magnitude of the hyperplasia may be somewhat smaller or larger than shown in this example, depending on the size. HELU, hyperplastic enlarged lobular unit; TDLU, terminal duct lobular unit.

Enlarged terminal duct lobular units (TDLUs) are common alterations in the adult female human breast. They are typically lined by crowded columnar epithelial cells, and the degree of enlargement can be substantial (Fig. 1). They have been discussed intermittently in the medical literature since the early 1900s, if not earlier, and some authors speculated as far back as then that they may have premalignant potential [1-3]. Consistent with this, a handful of recent studies [4-6] assigned a weak (about twofold) increased relative risk for developing breast cancer to enlarged lobules.

Because of the long and somewhat sporadic interest in this lesion, and because the histologic features of the epithelium lining the enlarged lobules may vary (e.g. size, shape, and atypia), it has been referred to by many terms over the years, including abnormal involution [1], columnar distention of acini [7], blunt duct adenosis [8], atypical lobules type A [9], columnar alteration of lobules [10], and columnar cell lesions with or without atypia [11] to name but a few. There has been considerable renewed interest in these lesions during the past few years, primarily because of their possible premalignant potential, which has resulted in even more diverse terminology as pathologists have struggled to define subtypes that encompass their histological variability [3,5,11-13].

From another perspective, this alteration can be viewed as a biological process in which the common unifying features are cellular hyperplasia resulting in enlargement of lobules. In this sense, we prefer the more general descriptive terminology 'hyperplastic enlarged lobular units', and the abbreviation HELU has symmetry with TDLU, from which HELUs evolve. Staying with a biological viewpoint, the histological diversity of HELUs can be seen as morphologic manifestations of variable differentiation and/or acquired genetic alterations in the cells.

Hyperplasia is defined as an increase in cell number that may result in the enlargement of a tissue [14]. It can be a physiologic or pathologic process, and the latter is often associated with delayed or altered differentiation. Growth and differentiation in normal breast epithelium are regulated by many biological mechanisms that include, in particular, the hormones estrogen and progesterone, whose effects are mediated through the estrogen receptor (ER)-α and progesterone receptor (PR), functioning as nuclear transcription factors [15,16]. In this study we examined correlations between growth (proliferation and apoptosis), endogenous hormone levels (using age as a surrogate for menopausal/estrogen status), and ER-α/PR expression in HELUs as compared with adjacent normal TDLUs in order to gain insight into the relative loss of growth control (i.e. hyperplasia) associated with the development of HELUs.

## Materials and methods

ER-α, PR, Ki67, apoptosis, and ER-α/Ki67 colocalization were assessed in formalin-fixed paraffin-embedded tissue samples of adult female human breast tissue from the Baylor College of Medicine affiliated hospitals, with institutional review board approval. The study population consisted of 324 total breasts and each biomarker was evaluated in large overlapping subsets of this population (because of their small size, not all TDLUs and HELUs could be evaluated for all factors).

ER-α was evaluated by immunohistochemistry in 615 TDLUs and 398 HELUs from 262 breasts, including 238 breasts containing at least one TDLU and one adjacent HELU. The immunohistochemical assay used mouse monoclonal antibody 6F11 (Novocastra, Newcastle, UK) specific for ER-α and a standard detection system as previously described [17]. ER-α expression was quantified by estimating the proportion of positive staining nuclei as previously described (0 = none, 1 = <1/100, 2 = 1/100 to 1/10, 3 = 1/10 to 1/3, 4 = 1/3 to 2/3, and 5 = >2/3) [17,18].

PR was assessed by immunohistochemistry in 647 TDLUs and 584 HELUs from 319 breasts, including 269 breasts with at least one TDLU and one adjacent HELU. The immunohistochemical assay used mouse monoclonal antibody 1294 (Dako, Carpinteria, CA, USA) against PR (A and B isoforms) [19] and a standard detection system as previously described [20]. PR expression was quantified in the same manner as ER-α.

Proliferation rate was assessed in 249 TDLUs and 137 HELUs from 114 breasts, including 81 breasts with at least one TDLU and one adjacent HELU. The immunohistochemical assay used mouse monoclonal antibody MiB1 (Dako) against the Ki67 proliferation-associated protein and a standard detection system as previously described [21]. The proliferation rate was quantified by absolute counting of positive and negative cells in each sample (average about 500 cells/sample) and expressed as percentage positive.

Apoptotic rate was assessed in 433 TDLUs and 396 HELUs from 165 breasts, including 133 breasts containing at least one TDLU and one adjacent HELU, using the terminal transferase-mediated dUTP nick end-labeling (TUNEL) assay as previously described [22]. Rates were quantified by absolute counting of positive and negative cells in each sample (average about 500 cells/sample) and expressed as percentage positive.

Colocalization of ER-α and Ki67 was assessed by dual-labeled immunofluorescence in 100 TDLUs and 100 HELUs from the same 25 breasts. ER-α was labeled with mouse monoclonal antibody 6F11 (Novocastra) and anti-mouse linking antibody conjugated to red fluorochrome Alexa 594 (Molecular Probes Inc., Eugene, OR, USA). Ki67 was labeled with rabbit polyclonal antibody Ki67 (Novocastra) and anti-rabbit linking antibody conjugated to green fluorochrome Alexa 488 (Molecular Probes). Colocalization was quantified by absolute counting of all Ki67 positive cells, which were either positive or negative for ER-α expression and expressed as percentage dual-positive cells relative to total Ki67 positive cells.

Comparisons of expression were performed using two-way, mixed model analysis of variance, with both fixed effects and random effect to account for the fact that some breasts contained both types of samples (i.e. TDLUs and HELUs) whereas others contained only one type, respectively. The linear model from the analysis of variance was used to compute least squares means and approximate standard errors for each group. Least square means estimate population means, balancing for uneven numbers of observations of TDLUs and HELUs within individual breasts. The term 'average' was used in place of 'least square means' throughout this manuscript to improve readability. Comparisons of age groups within sample types were accomplished with linear contrasts and analyses were performed with SAS software (version 9.1; SAS Institute Inc., Cary, NC).

## Results

### Receptor expression and growth

**Figure 2:**
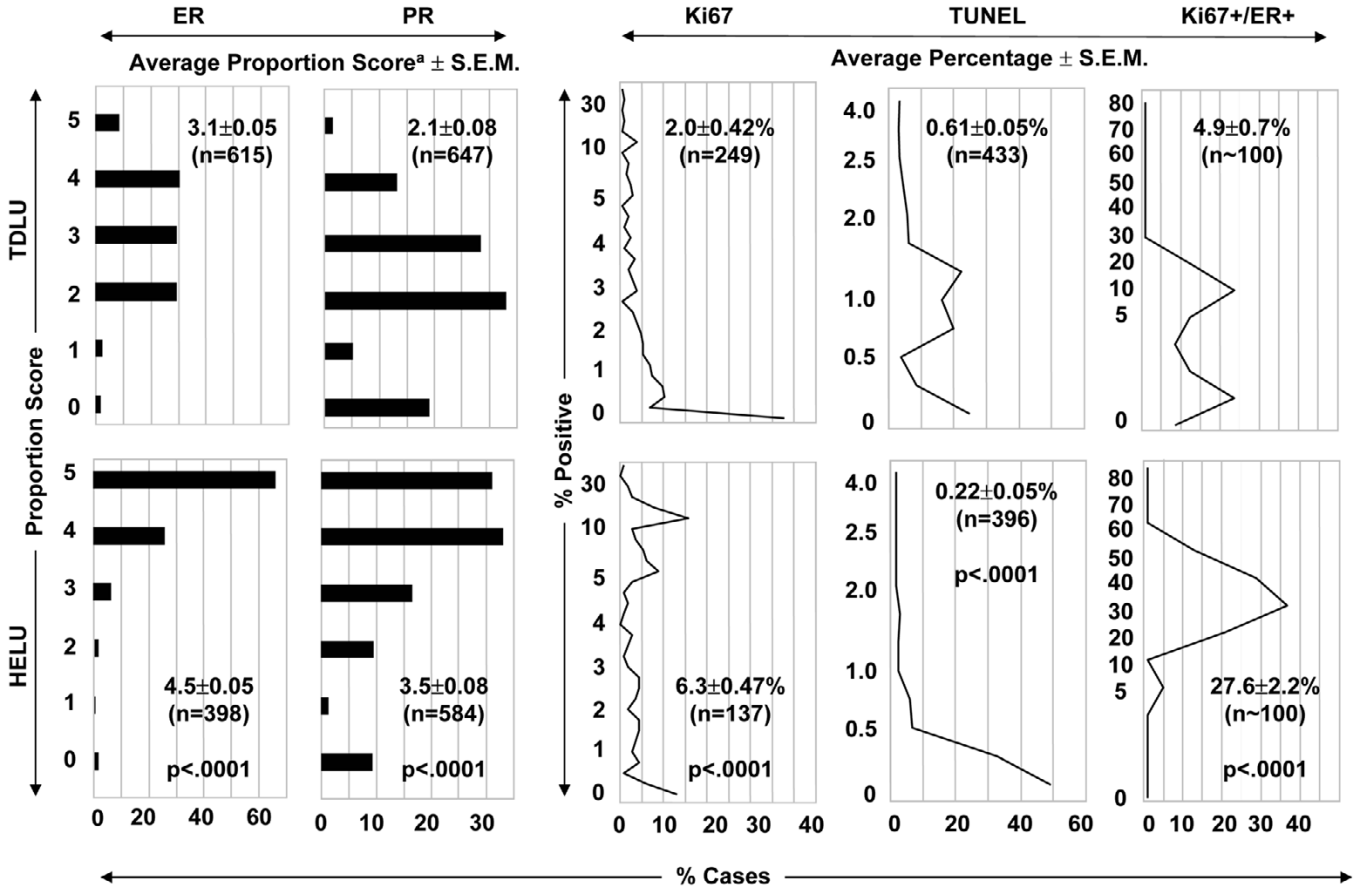
Receptor expression and growth. This figure compares normal TDLUs and HELUs for ER-α expression, PR expression, proliferation (Ki67 antigen), apoptosis (TUNEL assay), and colocalization of ER-α and Ki67 in the same cells. ^a^Average proportion score (± SEM) representing the estimated proportion of positive cells (0 = none, 1 < 1/100, 2 = 1/100 to 1/10, 3 = 1/10 to 1/3, 4 = 1/3 to 2/3, and 5 = >2/3). ER, estrogen receptor; HELU, hyperplastic enlarged lobular unit; PR, progesterone receptor; SEM, standard error of the mean; TDLU, terminal duct lobular unit; TUNEL, terminal transferase-mediated dUTP nick end-labeling.

**Figure 3:**
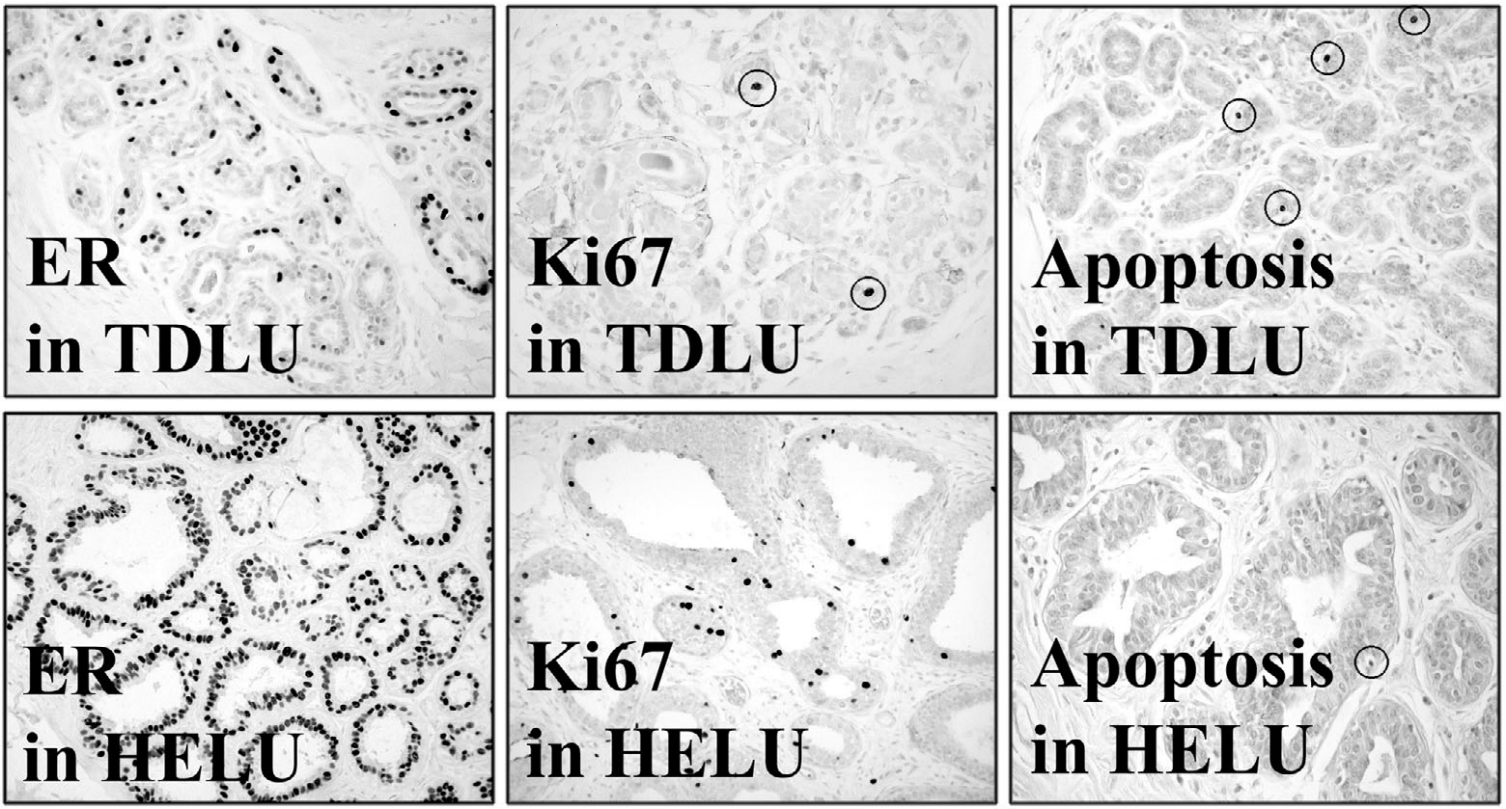
Examples of receptor expression and growth. This figure shows photomicrographs of representative examples of normal TDLUs and HELUs immunostained for ER-α, the proliferation-associated marker Ki67, and apoptosis (as assessed using the TUNEL assay). In general, HELUs exhibited much higher levels of ER-α expression than did TDLUs. Expression of progesterone receptor was similar to that of ER-α (not shown). Proliferation was also significantly higher on average in HELUs than in TDLUs. In contrast, apoptotic rates were somewhat lower in HELUs than in TDLUs. The dark (black) nuclei are positive for ER-α, Ki67 (some circled), and apoptosis (some circled) in each panel. ER, estrogen receptor; HELU, hyperplastic enlarged lobular unit; TDLU, terminal duct lobular unit; TUNEL, terminal transferase-mediated dUTP nick end-labeling.

ER-α expression, PR expression, proliferation rate (Ki67), apoptosis rate (TUNEL), and the percentage of proliferating/ER-α-positive cells are shown in Fig. 2. ER-α expression was significantly higher in HELUs than in TDLUs, with proportion scores averaging 4.5 ± 0.05 and 3.1 ± 0.05, respectively (*P *< 0.0001). These values roughly correspond to about 85% and 35% average positive cells, but the scores are nonlinear estimates of proportion and difficult to convert to exact percentages. Similarly, PR expression was significantly higher in HELUs than in TDLUs, with proportion scores averaging 3.5 ± 0.08 and 2.1 ± 0.08 (*P *< 0.0001), respectively, corresponding to roughly 50% and 15% positive cells. ER-α and PR expression were significantly higher in HELUs than in adjacent TDLUs in 85% of breasts containing both types of samples, whereas expression levels were similar in the remaining 15% of breasts (detailed data not shown). Proliferation was also significantly elevated in HELUs as compared with TDLUs, averaging 6.3 ± 0.47% and 2.0 ± 0.42%, respectively (*P *< 0.0001). In contrast, apoptosis was significantly lower in HELUs than in TDLUs, averaging 0.22 ± 0.05% and 0.61 ± 0.05% (*P *< 0.0001). On average, only 4.9 ± 0.7% of proliferating normal epithelial cells in TDLUs simultaneously expressed ER-α, compared with 27.6 ± 2.2% in HELUs, which was also a highly significant difference (*P *< 0.0001). Figure 3 shows representative photomicrographs of ER-α expression, proliferation, and apoptosis in HELUs and TDLUs.

### Changes in receptors and growth with menopausal status

Table 1 shows the relationships between ER-α expression, PR expression, proliferation (Ki67), and apoptosis (TUNEL) in TDLUs compared with HELUs in the same breasts stratified by patient age as a surrogate for menopausal/estrogen status. ER-α was significantly lower in TDLUs and HELUs in premenopausal than in postmenopausal breasts, with average proportion scores of 2.65 versus 3.62 (approximately 25% versus 55%; *P *< 0.0001) and 4.37 versus 4.66 (approximately 75% versus 90%; *P *= 0.013), respectively. PR was higher in premenopausal than in postmenopausal TDLUs and HELUs, with average proportion scores of 2.15 versus 2.01 (about 20% versus 10%; *P *= 0.08) and 3.58 versus 3.34 (about 60% versus 45%; *P *= 0.02), respectively. The rates of proliferation were higher in premenopausal than in postmenopausal TDLUs and HELUs, averaging 2.52% versus 1.38% (*P *= 0.024) and 7.32% versus 5.11% (*P *= 0.017), respectively. Apoptosis was significantly higher in TDLUs from premenopausal than from postmenopausal breasts, averaging 0.80% and 0.43% (*P *< 0.0001). In contrast, apoptosis was statistically similar in premenopausal and postmenopausal HELUs (0.26% versus 0.18%; *P *= 0.38).

## Discussion

Enlarged TDLUs, by any name, are a common alteration in the adult female human breast. Although they have no well established clinical significance, they have attracted considerable attention recently, primarily because they may represent a very early potential precursor of breast cancer – a suggestion that dates as far back as the early 1900s [1-3]. The majority are lined by a single layer of hyperplastic columnar epithelial cells with minimal nuclear atypia, but a substantial minority exhibit more diverse histologic features such as stratification of cells, apocrine-like secretory features, and significant nuclear atypia, contributing to the complex terminology that has evolved to describe them [3,5,11-13].

More than 30 years ago, in one of the most enlightening papers on this issue, Wellings and Jensen [9] described a '... continuum of morphologic structures linking normal terminal ductal lobular units ... to the entire group of dysplastic, metaplastic, hyperplastic, anaplastic, and neoplastic human mammary lesions ...'. Enlarged TDLUs, which they referred to as atypical lobules type A (and we refer to as HELUs), played a central role in this continuum as an early 'hyperplastic' departure from normal TDLUs with the ability to develop or progress into several other types of lesions in the breast, including atypical ductal hyperplasia (ADH). ADH is widely regarded as an unequivocal but nonobligatory precursor of ductal carcinoma *in situ *(DCIS), which in turn is considered to be a relatively committed progenitor of invasive breast cancer (primarily the so-called 'ductal' invasive breast cancers, which account for about 80% of all invasive breast cancers). In this sense, HELUs can be viewed as the earliest histologically identifiable potential precursor of breast cancer. In Wellings' and Jensen's words [9,23], '... atypical lobules type A ... showed variable degrees of anaplasia forming [a] continuum from normal lobules to ductal carcinoma *in situ* ...'.

**Figure 4:**
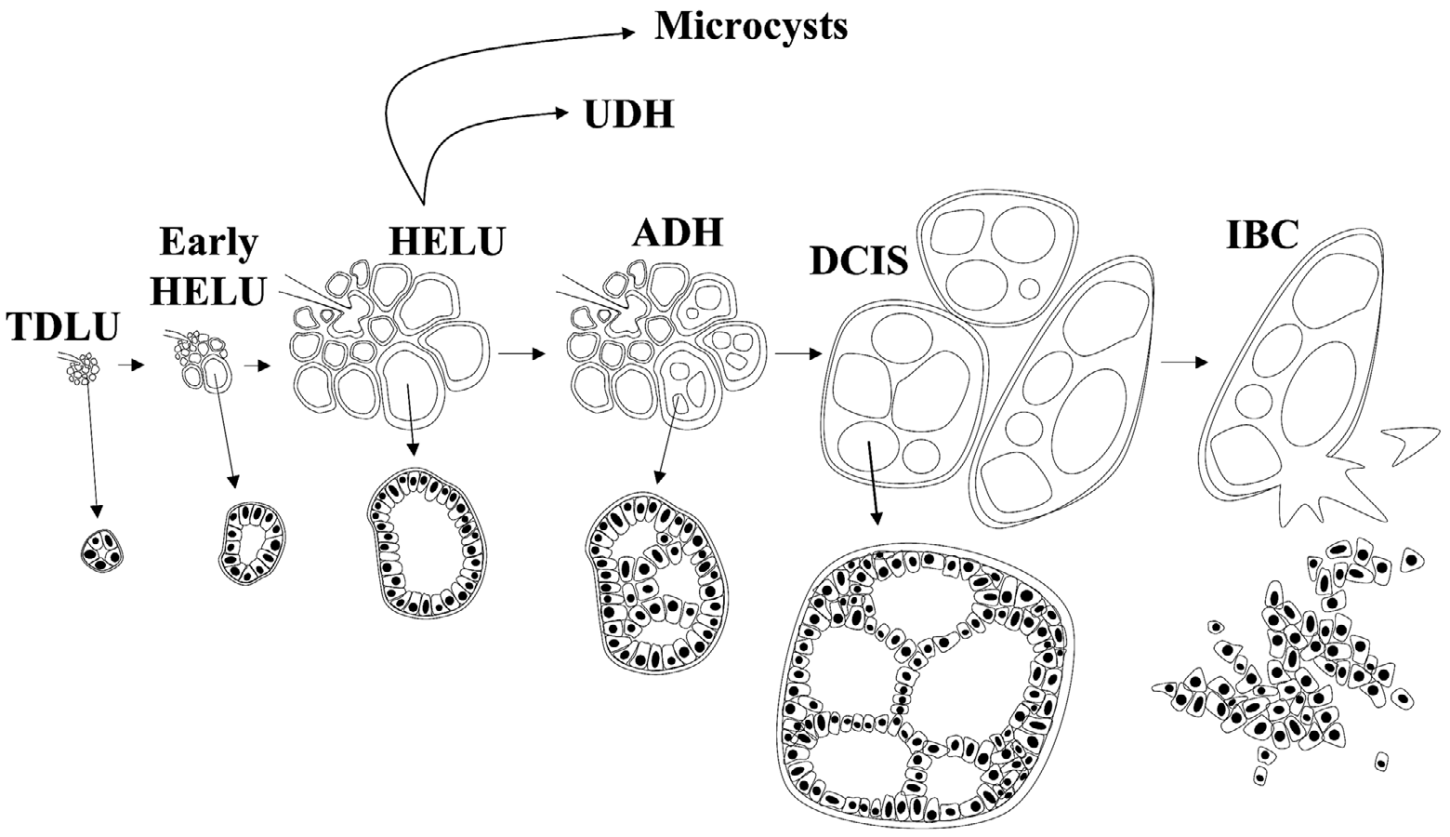
Modified Wellings Jensen model of breast cancer evolution. This figure illustrates our interpretation of the model of 'ductal' breast cancer evolution originally proposed by Wellings and Jensen [9], which was based almost entirely on the evidence of histologic continuity. In this model, hyperplastic breast epithelial cells gradually enlarge normal TDLUs to form HELUs. HELUs may then differentiate to microcysts (often with apocrine features) or progress to more complex lesions including UDH and ADH. ADH may progress to DCIS as the cells continue to proliferate and distend the acini, and DCIS may eventually progress to IBC. Progression is probably very slow overall and nonobligatory, so, for example, only a small subset of HELUs ever progress to ADH or beyond. Whether and how differentiation and progression proceed is probably dictated by the acquisition of specific genetic and epigenetic abnormalities in a largely random manner. ADH, atypical ductal hyperplasia; DCIS, ductal carcinoma *in situ*; HELU, hyperplastic enlarged lobular unit; IBC, invasive breast cancer; TDLU, terminal duct lobular unit; UDH, usual ductal hyperplasia.

**Figure 5:**
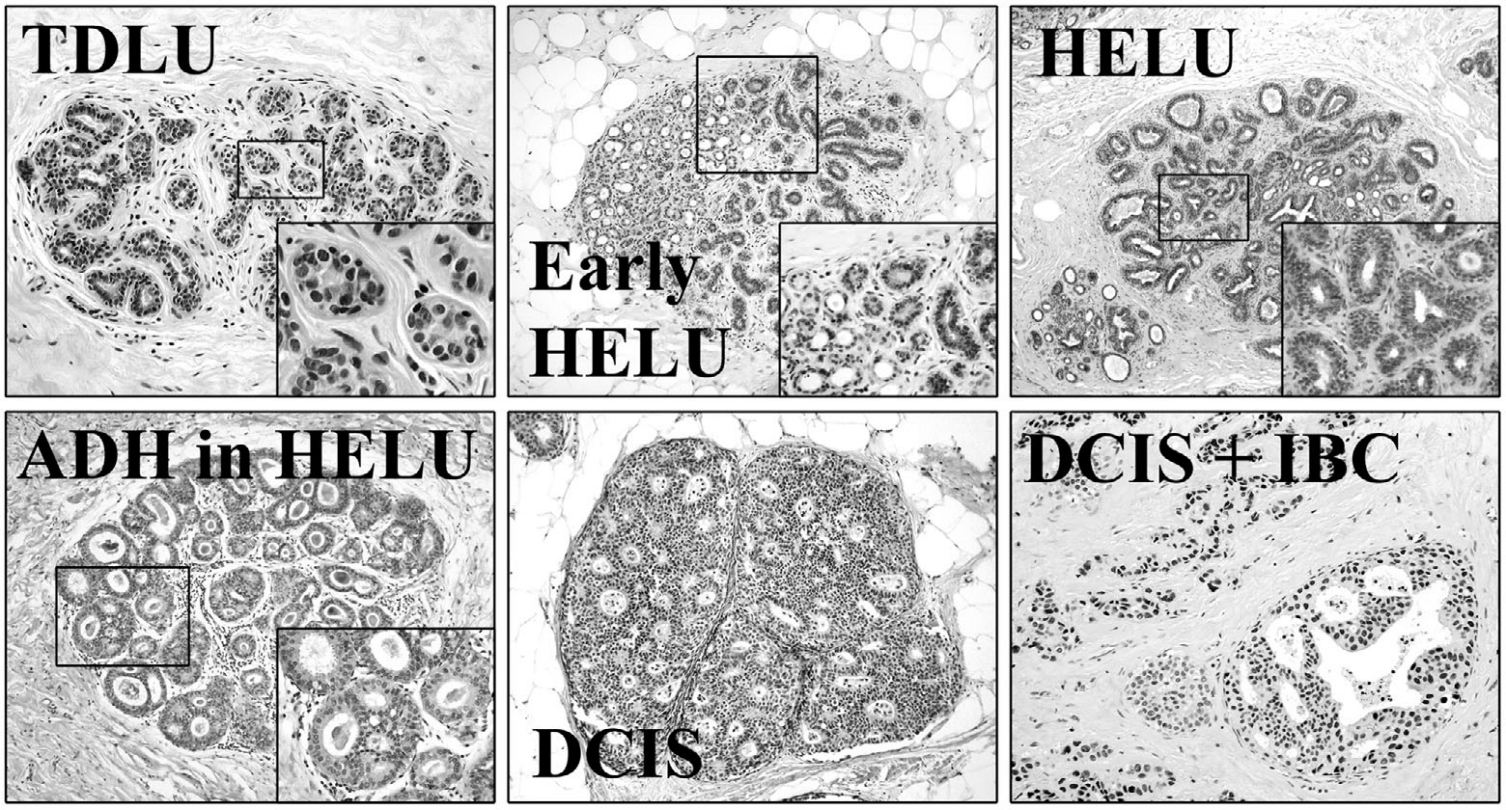
Stages of breast cancer evolution. This figure shows representative photomicrographs of important artificially defined stages of the Wellings–Jensen model of 'ductal' breast cancer evolution, including normal TDLUs, HELUs, ADH, DCIS, and IBC. ADH, atypical ductal hyperplasia; DCIS, ductal carcinoma *in situ*; HELU, hyperplastic enlarged lobular unit; IBC, invasive breast cancer; TDLU, terminal duct lobular unit.

The Wellings and Jensen model was based almost entirely on the evidence of gradual histologic continuity observed between thousands of samples of normal and abnormal human breast tissues examined grossly and microscopically, and it was consistent with previous observations in mouse models of breast cancer that they later extended to humans [9]. We have confirmed these observations and found the model to be very compelling, with minor modifications of our own (Figs 4 and 5). Gradual histologic continuity of this magnitude is persuasive evidence and is as close as possible to observing tumor progression in humans. In addition, the model has withstood the test of time in the sense that it remains consistent with newer discoveries such as the escalating risk for developing breast cancer associated with HELUs (about twofold), ADH (about fivefold), and DCIS (about 10-fold) [5,6,24-29], and the shared genetic alterations between them, especially when they occur in the same breasts [30-34].

In our view, progression in this model is probably very slow overall and nonobligatory so, for example, only a small subset of HELUs may ever progress to ADH or beyond. Whether and how progression proceeds is probably dictated by the accumulation of specific genetic and epigenetic abnormalities in a largely random manner. We doubt that the artificially defined sequential stages in this morphologic model represent the only pathway to breast cancer, but it is probably a very important one because the histologic continuity is so strong, in addition to the epidemiologic and genetic evidence already mentioned. There are many other types of benign proliferative lesions in the breast that, for the most part, do not exhibit substantial histologic continuity with breast cancer; therefore, if other pathways to cancer exist, then they must be histologically subtle, perhaps occurring at the level of individual cells or too rapidly to be easily observed.

The growth and differentiation of normal breast epithelial cells lining TDLUs are regulated by many biologic mechanisms, especially one involving circulating estrogen, which binds and activates the nuclear transcription factor ER-α [16,35]. Among many functions, activated ER-α regulates the synthesis of PR, another nuclear transcription factor that, in response to progesterone, also helps to regulate growth and differentiation in normal cells [16,35,36]. The present study evaluated and contrasted ER-α, PR, and growth (proliferation and apoptosis) in TDLUs and adjacent HELUs to gain insight into the relative loss of growth control (i.e. hyperplasia) leading to development of the latter.

Consistent with earlier studies [33], we observed that nearly all TDLUs contained ER-α-positive cells, averaging about 35%. Using age as a surrogate for menopausal and estrogen status, expression in TDLUs was significantly lower in premenopausal than in postmenopausal breasts, which has previously been reported [33] and which is consistent with the normal negative regulation of ER-α by estrogen [16]. In contrast, about 85% of cells in HELUs expressed ER-α; this is much higher than in TDLUs and is consistent with another recent report of elevated ER-α in HELUs [34]. Similar to TDLUs, ER-α expression in HELUs was significantly lower in premenopausal than in postmenopausal breasts, suggesting that hyperplastic cells remain responsive to regulation by estrogen, although to a lesser degree than normal cells do. It will be important to determine the fundamental causes for the large elevation in ER-α in HELUs. Overall, the findings for PR were similar to those for ER-α, with the exception that PR levels were relatively higher in premenopausal samples, which is consistent with normal induction of PR by estrogen-activated ER-α [16,36].

Estrogen and progesterone are potent growth factors for normal breast epithelium [15,16,35,36], which is consistent with our observation that average proliferation was significantly higher in TDLUs from premenopausal than in postmenopausal breasts, which has also been observed by others [33,35]. A reasonable hypothesis for the development and growth of HELUs is increased proliferation due to large increases in ER-α and PR, which are the receptors for these mitogens. Consistent with this, we observed a significant (more than threefold) increase in average proliferation in HELUs compared with adjacent TDLUs. A recent study [37] which transfected and over-expressed murine ER-α in the epithelium of mouse mammary glands noted the rapid development of hyperplasias, which occasionally progressed to DCIS; this supports the notion that elevated ER-α may be partially responsible for the development of HELUs and their progression to more advanced lesions in humans. However, a substantial (about 15%) subset of HELUs did not exhibit elevated ER-α, suggesting that other mechanisms may also be involved.

In our study, the range of proliferation rates in HELUs was wide (0–36%), exhibited significant overlap with TDLUs, and was somewhat bimodal. Proliferation at the low end of this bimodal distribution averaged only about 2% (similar to TDLUs) but was about 15% at the high end (far above TDLUs), and perhaps a subset of the latter group of HELUs is more likely to progress to lesions with higher breast cancer risk such as ADH.

In normal breast, proliferating cells are almost always (>95%) ER-α negative; conversely, ER-α positive cells are rarely (<5%) proliferating [35,38,39]. It is currently unknown whether this reflects separate populations of stable cells or the same population in which receptor expression and proliferation vary with time (e.g. during the cell cycle). Because hormones (especially estrogen) are required for proliferation, the latter hypothesis is plausible, but the former could be explained by paracrine signaling between stable ER-α-positive cells promoting proliferation in nearby stable ER-α-negative cells, and these possibilities are not mutually exclusive [15,35]. Regardless, estrogen is necessary to stimulate proliferation in normal cells. Compared with TDLUs, HELUs exhibited a more than fivefold increase in the average percentage of ER-α-positive proliferating cells (4.9% versus 27.6% overall), suggesting that the hormonal regulation of proliferation in these cells is altered and/or that other pathways have been activated that stimulate proliferation independent of ER-α expression. A similar increase in ER-α-positive proliferating cells has previously been reported in more advanced precursors (e.g. ADH and DCIS) and invasive breast cancer [35,38,39], which is consistent with the hypothesis that HELUs represent an earlier stage of the same continuum.

In mice, as in humans, ER-α-positive/proliferating normal epithelial cells are relatively rare, and a recent study in mice [40] suggested that they may represent important mammary progenitor cells in which proliferation is inhibited by autocrine signaling through transforming growth factor (TGF)-β_1_, resulting in a normally large population of ER-α-positive/nonproliferating cells. The study further showed that when TGF-β_1 _signaling is inhibited, the proportion of ER-α-positive/proliferating cells increases dramatically, reaching the levels we observed in HELUs. Therefore, HELUs perhaps represent an expanding population of precursor cells resulting from delayed differentiation associated with decreased TGF-β_1 _signaling, and it will be important to assess the status of TGF-β_1 _in HELUs.

The rate of cell death also contributes to the overall growth of cellular tissues. We observed a relatively low average rate of apoptosis in normal TDLUs (about 0.61%), which was significantly higher (about twofold) in premenopausal than postmenopausal breasts, which is consistent with normal estrogen regulation of programmed cell death in the breast [41]. In contrast, apoptosis was significantly lower in HELUs (about 0.22%), and it did not fluctuate significantly with menopausal status, suggesting that estrogen regulation of apoptosis may be abnormal.

## Conclusion

HELUs are a common alteration of normal TDLUs and represent the earliest histologically identifiable potential precursor of breast cancer. The prominent hyperplasia leading to the development of HELUs is associated with both increased proliferation and decreased cell death relative to normal cells. ER-α and PR are highly elevated in HELUs, which may partially explain this hyperplasia because they are the receptors for the hormones that regulate proliferation and apoptosis, although additional mechanisms are likely to be involved. Future studies will hopefully reveal the fundamental causes of increased receptor expression and growth in HELUs, which may lead to better prediction of breast cancer risk and new strategies for preventive therapy.

## Abbreviations

ADH = atypical ductal hyperplasia; DCIS = ductal carcinoma *in situ*; ER = estrogen receptor; HELU = hyperplastic enlarged lobular unit; IBC = invasive breast cancer; PR = progesterone receptor; TDLU = terminal duct lobular unit; TGF = transforming growth factor; TUNEL = terminal transferase-mediated dUTP nick end-labeling.

## Competing interests

The authors declare that they have no competing interests.

## Authors' contributions

SL participated in designing the study, acquiring data (immunohistochemistry), interpreting data, and outlining and reviewing the manuscript. SKM participated in designing the study, acquiring study samples, acquiring data (immunohistochemistry), interpreting data, and outlining and reviewing the manuscript. SM participated in acquiring study samples, acquiring data (immunohistochemistry and immunofluorescence), interpreting data, and reviewing the manuscript. SGH participated in designing the study, analyzing data (statistical analyses), and reviewing the manuscript. DM participated in designing the study, acquiring data (immunofluorescence), analyzing data, and outlining and reviewing the manuscript. DCA participated in designing the study, acquiring study samples, acquiring data (immunohistochemical and immunofluorescence), analyzing data, and preparing the manuscript.

## Figures and Tables

**Table 1:** Changes in receptors and growth with menopausal status

Marker	Age ≤50 years (mean ± SEM)	Age >50 years (mean ± SEM)	*P *value
ER-α-TDLU^a^	2.65 ± 0.06 (*n *= 342)	3.62 ± 0.06 (*n *= 273)	<0.0001
ER-α-HELU^a^	4.37 ± 0.07 (*n *= 201)	4.66 ± 0.07 (*n *= 197)	0.013
PR-TDLU^a^	2.15 ± 0.09 (*n *= 383)	2.01 ± 0.10 (*n *= 264)	0.08
PR-HELU^a^	3.58 ± 0.10 (*n *= 274)	3.34 ± 0.10 (*n *= 236)	0.02
Ki67-TDLU	2.52 ± 0.67 (*n *= 106)	1.38 ± 0.69 (*n *= 95)	0.024
Ki67-HELU	7.32 ± 0.74 (*n *= 67)	5.11 ± 0.73 (*n *= 69)	0.017
TUNEL-TDLU	0.80 ± 0.06% (*n *= 234)	0.43 ± 0.07% (*n *= 142)	<0.0001
TUNEL-HELU	0.26 ± 0.06% (*n *= 215)	0.18 ± 0.07% (*n *= 140)	0.38

This table compares average ER-α expression, PR expression, proliferation rate (Ki67), and apoptosis rate (TUNEL) between normal TDLUs and HELUs from the same breasts, stratified by age as a surrogate for menopausal/estrogen status. ^a^Average proportion score representing the estimated proportion of positive cells (0 = none, 1 < 1/100, 2 = 1/100 to 1/10, 3 = 1/10 to 1/3, 4 = 1/3 to 2/3, and 5 = >2/3). ER, estrogen receptor; HELU, hyperplastic enlarged lobular unit; TDLU, terminal duct lobular unit; PR, progesterone receptor; TUNEL, terminal transferase-mediated dUTP nick end-labeling.
